# Supplementary figures and images for: Structure of the connexin-43 gap junction channel in a putative closed state
Source: eLife. 2023 Aug 3;12:RP87616. doi: 10.7554/eLife.87616 (PMC10400079; doi:10.7554/eLife.87616)

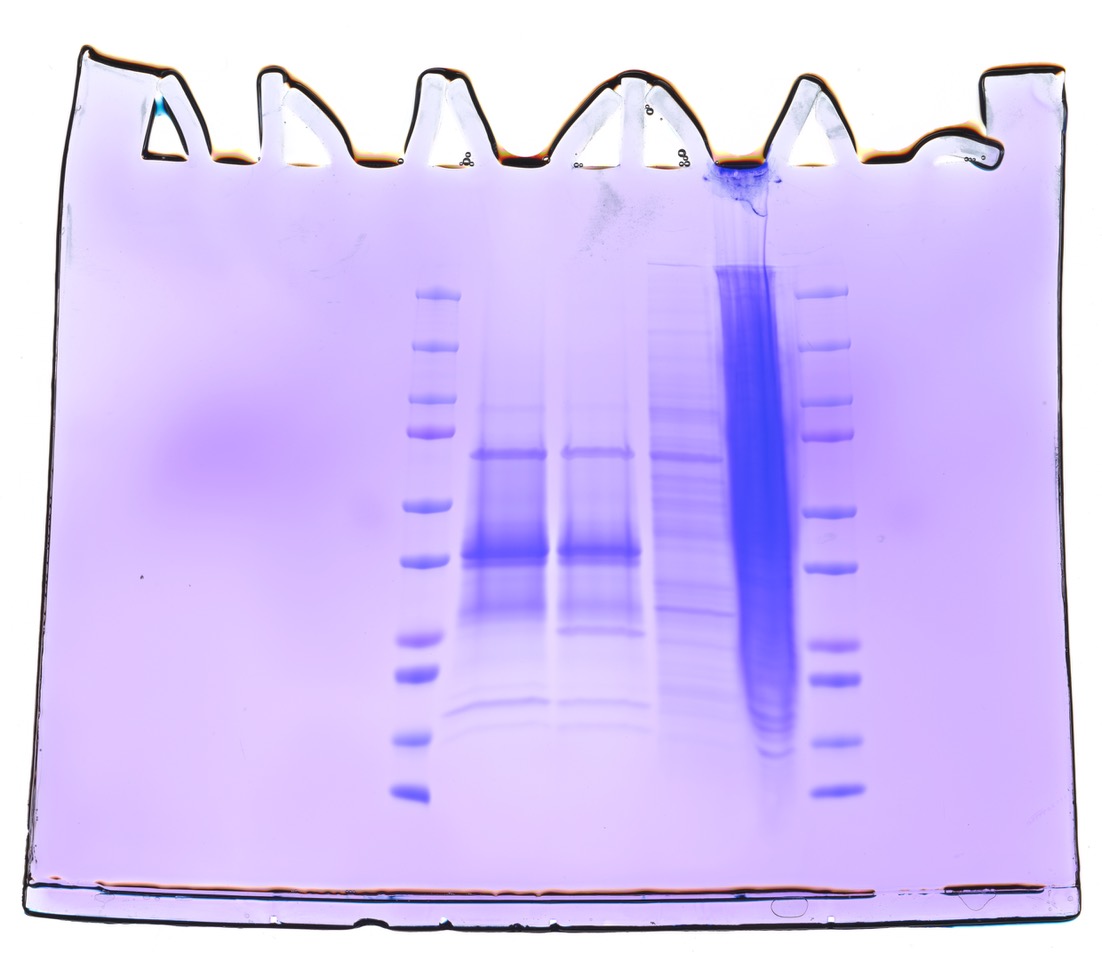

Supplement: Figure 1—figure supplement 2—source data 2. — A Coomassie blue-stained sodium dodecyl sulfate–polyacrylamide gel electrophoresis (SDS–PAGE) gel of connexin-43 (Cx43). [file elife-87616-fig1-figsupp2-data2.zip › Figure 1 - figure supplement 2 - source data 2.jpeg]

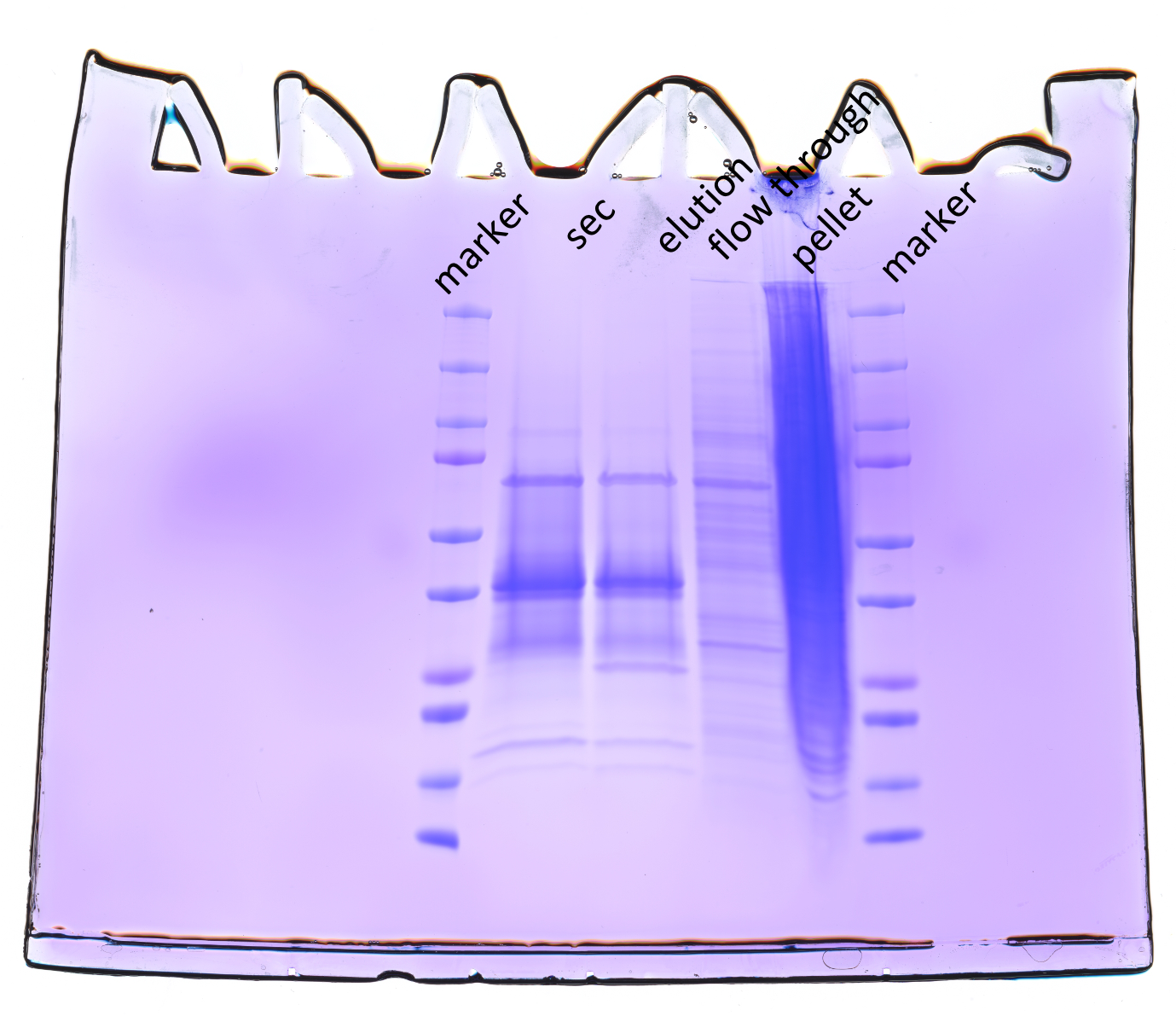

Supplement: Figure 1—figure supplement 2—source data 3. — A Coomassie blue-stained sodium dodecyl sulfate–polyacrylamide gel electrophoresis (SDS–PAGE) gel of connexin-43 (Cx43), with individual lanes labelled. [file elife-87616-fig1-figsupp2-data3.zip › Figure 1 - figure supplement 2 - source data 3.png]

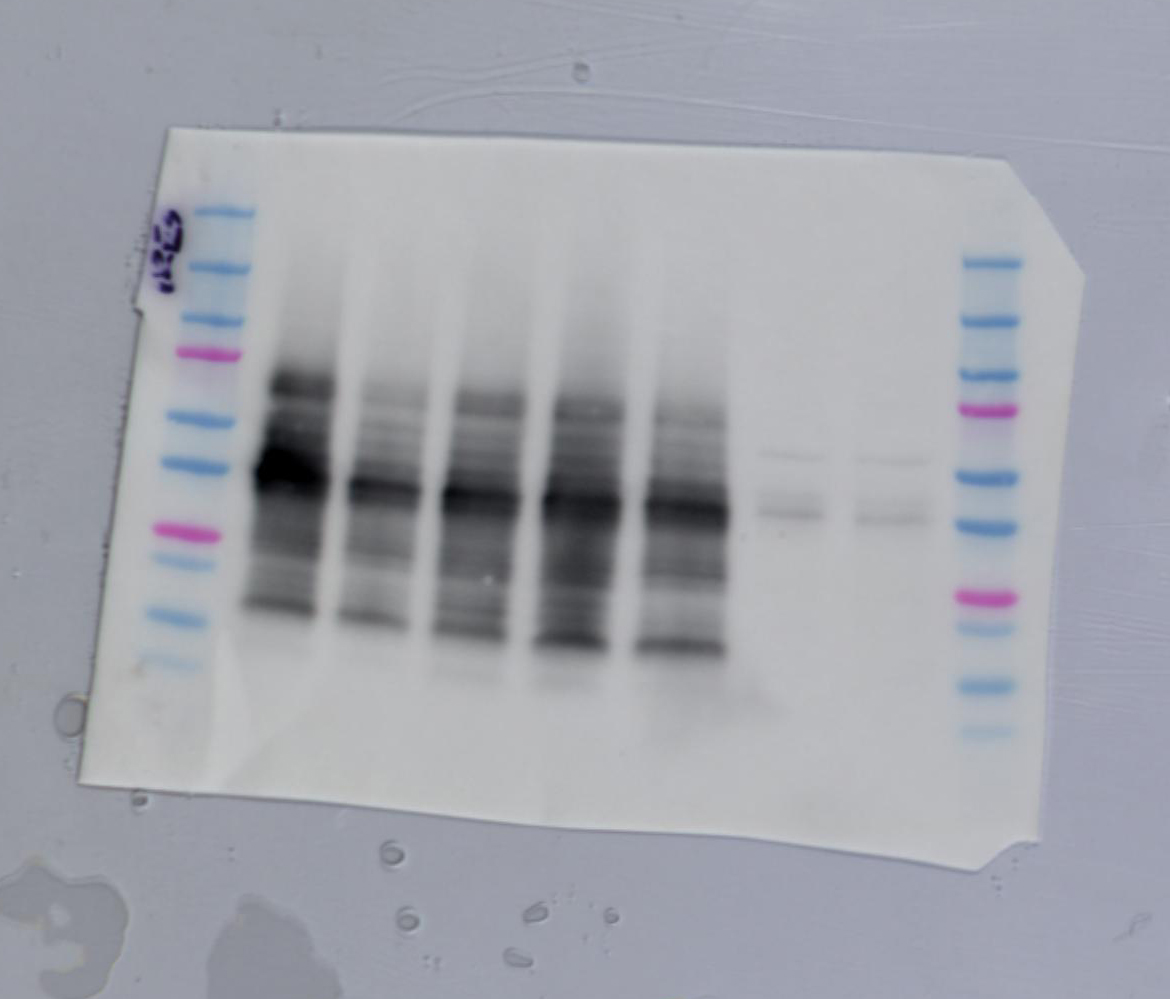

Supplement: Figure 1—figure supplement 2—source data 4. — Western blot of connexin-43 (Cx43)-expressing cell lysates (anti-Cx43 antibody). [file elife-87616-fig1-figsupp2-data4.zip › Figure 1 - figure supplement 2 - rouce data 4.jpg]

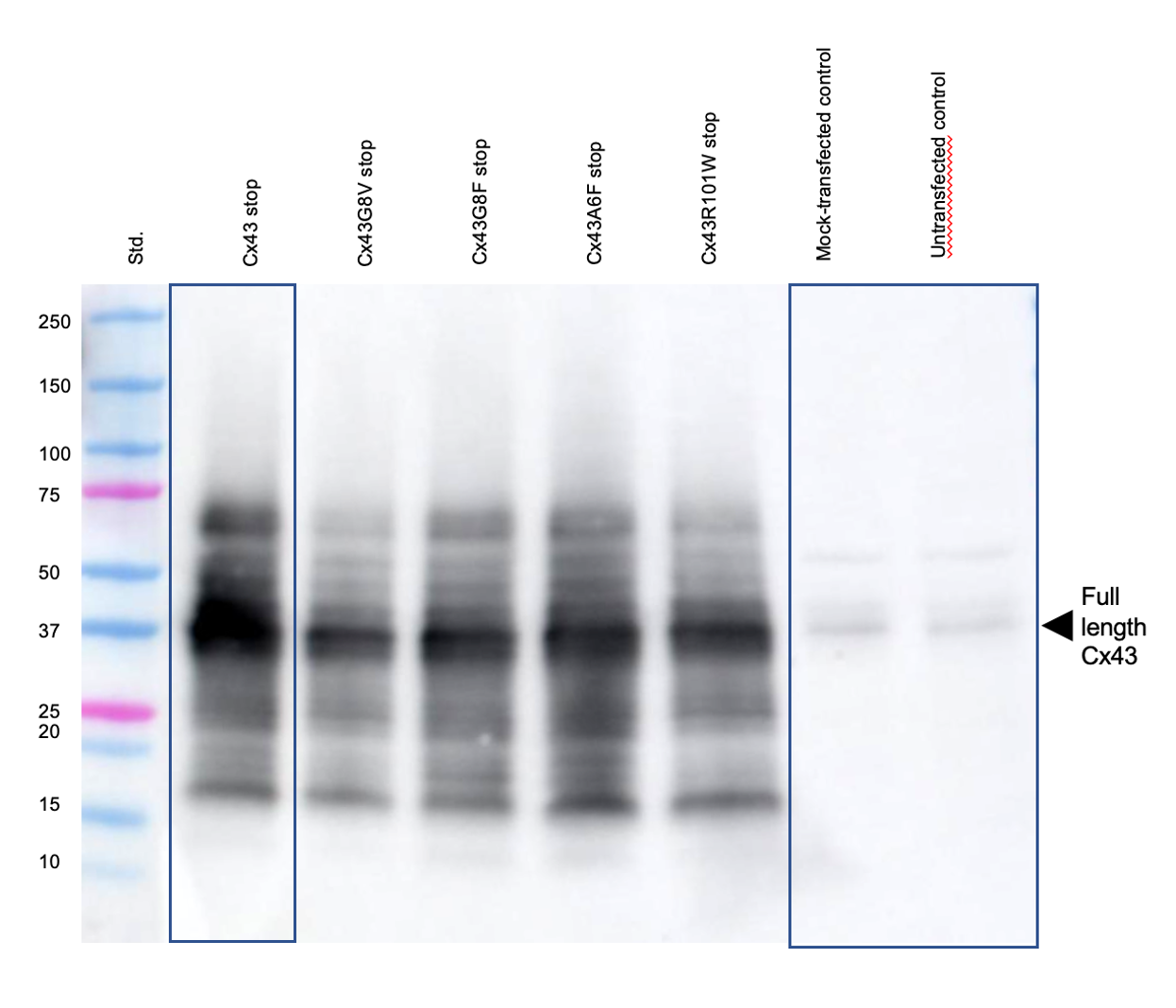

Supplement: Figure 1—figure supplement 2—source data 5. — Western blot of connexin-43 (Cx43)-expressing cell lysates (anti-Cx43 antibody), with lanes labelled and areas used in Figure 1—figure supplement 2D marked using boxes. [file elife-87616-fig1-figsupp2-data5.zip › Figure 1 - figure supplement 2 - rouce data 5.png]

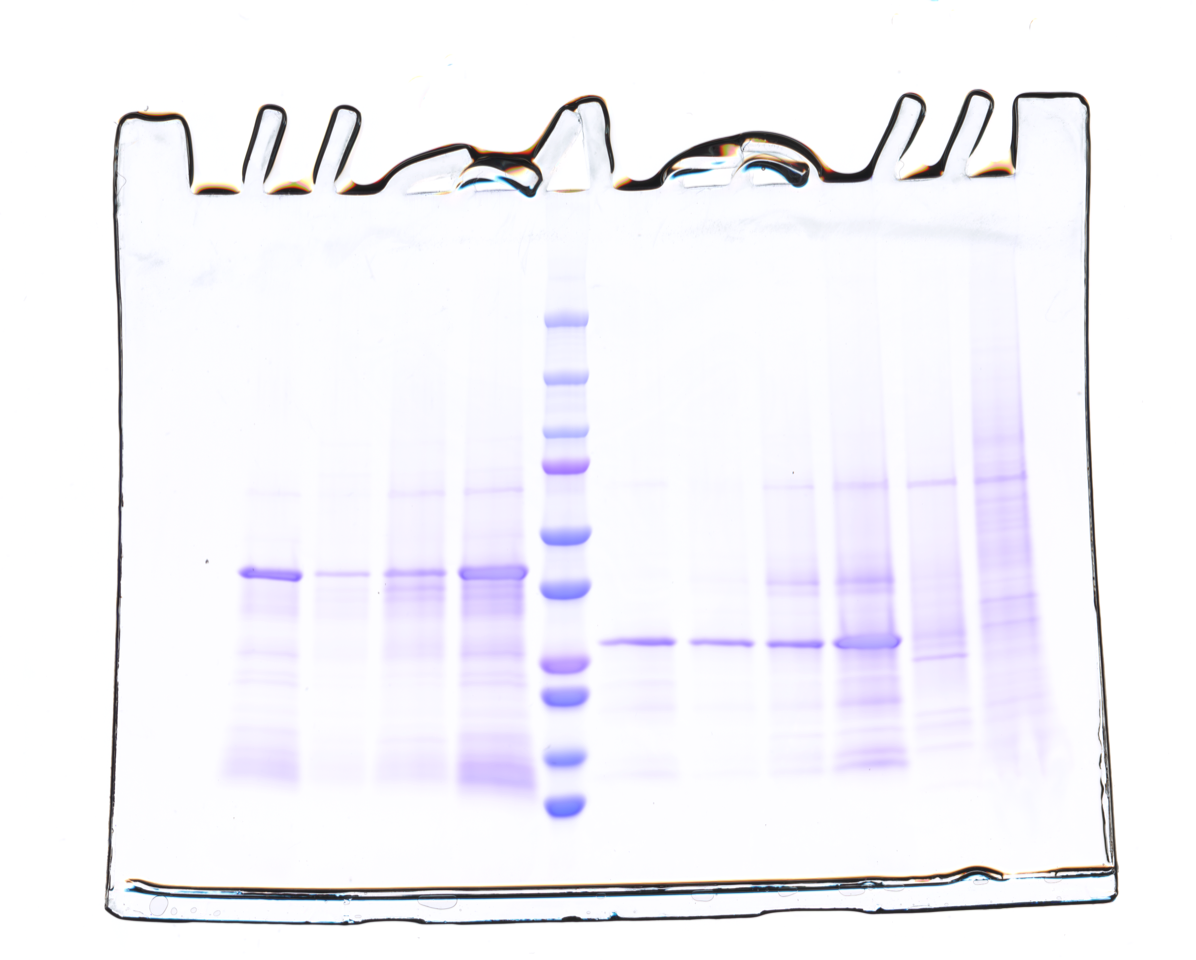

Supplement: Figure 1—figure supplement 2—source data 7. — A Coomassie blue-stained sodium dodecyl sulfate–polyacrylamide gel electrophoresis (SDS–PAGE) gel of connexin-43 (Cx43) reconstituted in MSP2N2 nanodiscs. [file elife-87616-fig1-figsupp2-data7.zip › Figure 1 - figure supplement 2 - source data 7.tiff]

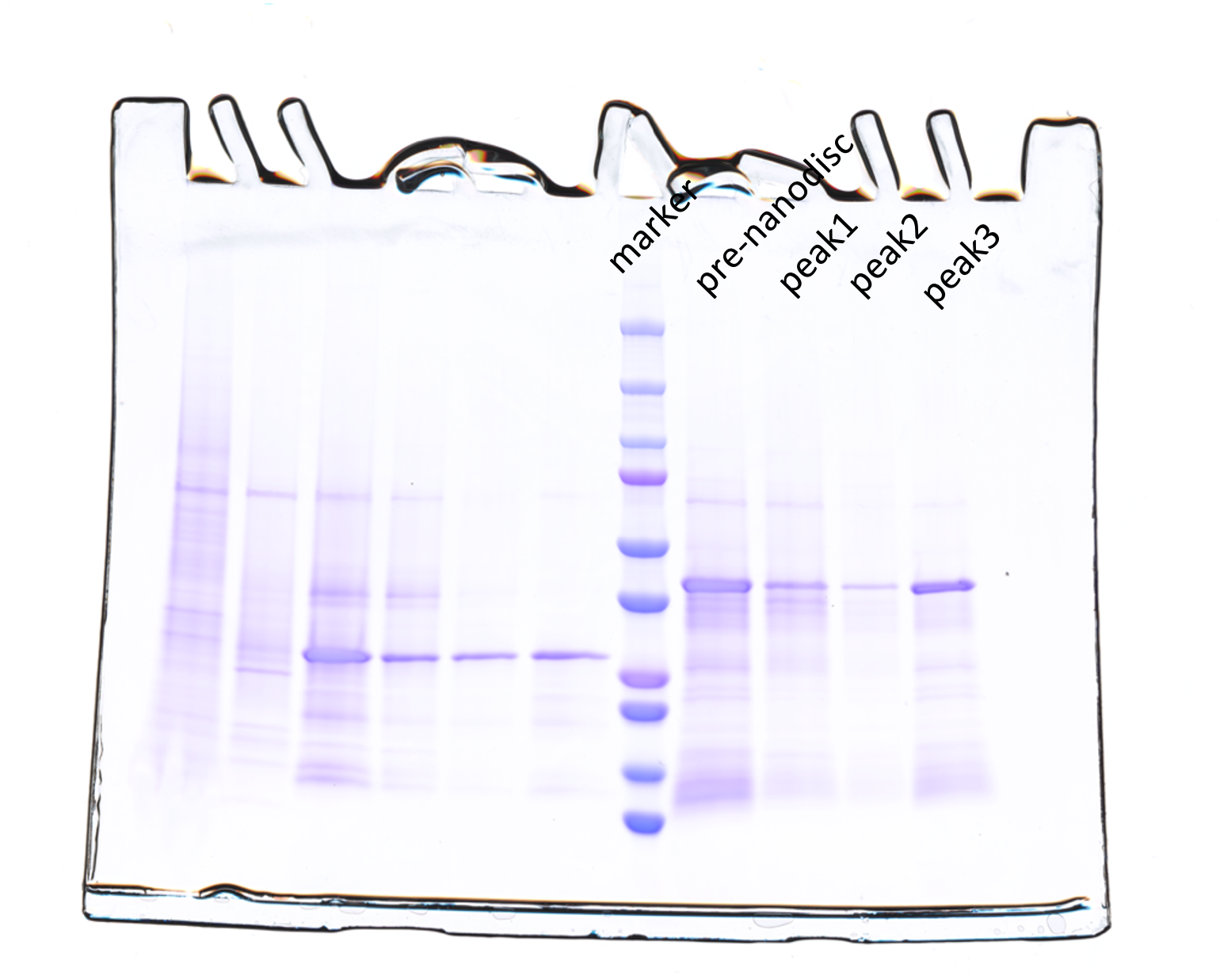

Supplement: Figure 1—figure supplement 2—source data 8. — A Coomassie blue-stained sodium dodecyl sulfate–polyacrylamide gel electrophoresis (SDS–PAGE) gel of connexin-43 (Cx43) reconstituted in MSP2N2 nanodiscs, with individual lanes labelled. [file elife-87616-fig1-figsupp2-data8.zip › Figure 1 - figure supplement 2 - source data 8.png]
